# Changes in Menopause Stage, Body Mass, and Fat Mass as Drivers of Midlife Changes in C-reactive Protein Levels: A Prospective Cohort Study

**DOI:** 10.21203/rs.3.rs-10924459/v1

**Published:** 2026-09-29

**Authors:** Arun S Karlamangla, Wei Juan Han, Duncan Thomas, Samar El Khoudary, Sherri-Ann Burnett-Bowie, Carrie Karvonen-Gutierrez, Sybil Crawford, Gail Greendale

**Affiliations:** University of California, Los Angeles; University of California, Los Angeles; Duke University; Virginia Commonwealth University; Harvard University; University of Michigan; University of Massachussetts; University of California, Los Angeles

**Keywords:** Menopause transition, serum C-reactive protein, midlife trajectory, body mass index, fat mass index, multi-ethnic cohort, fixed effects analysis

## Abstract

**Background.:**

Serum levels of C-reactive protein (CRP), a marker of chronic inflammation, increase in many, but not all, women going through the menopause transition (MT). It is not known whether this is driven entirely by changes in body mass and fat mass in midlife or by other physiological changes during the MT.

**Methods.:**

We use individual fixed effects analysis of repeated measurements across the MT, in a multi-ethnic cohort of 1681 women followed for over two decades (1996–2018), to address this knowledge gap; we also test whether associations between body mass increase (likewise fat mass increase) and CRP increase differ by race/ethnicity.

**Results.:**

Adjusted for changes in body mass index (BMI), change in menopause stage was not associated with change in CRP. Every kg/m^2^ within-woman increase in BMI was associated with 16% increase in serum CRP (p<.0001). Similarly, every kg/m^2^ within-woman increase in fat mass index (total fat mass divided by square of height, FMI) was associated with 23% increase in serum CRP (p<.0001). The association of BMI increase with CRP increase was smaller in Black than in White women: 12% vs. 18% per kg/m^2^ (p = 0.03 for test of difference), but not statistically different in Chinese and Japanese women relative to White women. On the other hand, race/ethnicity did not modify the association of FMI increase with CRP increase, but the association was modestly smaller in natural and surgical postmenopause than in premenopause: 20% and 15% vs. 22% per kg/m^2^, respectively (p = 0.001 and 0.05 for tests of difference).

**Conclusions.:**

Midlife increases in CRP levels in women are not directly related to menopause stage but appear to be driven by increases in body mass, particularly fat mass. While changes in body mass have different implications for CRP changes in Black vs. White women, increases in fat mass have similar associations with CRP increases in Black and White women. However, changes in fat mass have modestly smaller associations with CRP changes in postmenopause than before the MT.

## Background

Chronic sub-clinical inflammation, as indexed by elevated serum levels of C -reactive protein (CRP) measured by high-sensitivity assays, has been linked to a variety of poor health outcomes in multiple longitudinal studies [1–3], and to increased mortality from these health conditions [4,5].

CRP production by hepatocytes and macrophages is stimulated by inflammatory cytokines like interleukin (IL)-6, and adipose tissue is a major source of IL-6 [6]. In addition, adipocytes themselves produce CRP in response to cytokines [7]; it is therefore, not surprising that measures of obesity, such as body mass index (BMI) and total fat mass, correlate strongly with serum CRP levels in cross-sectional studies in both men and women [8–10], starting in early midlife [11]. There is a suggestion in these cross-sectional analyses that the association of BMI with CRP is stronger in men than in women [8], while the association of fat mass with CRP is stronger in women [12].

However, serum CRP levels are affected by heritable traits and specific genotypes [13,14]. Other potential confounders in cross-sectional studies of the obesity-CRP relationship are person-to-person differences in health behaviors (e.g., smoking, regular physical activity, meditation), levels of perceived stress - both financial and emotional, experiences of discrimination, and use of anti-inflammatory medications and statins [9,15–20]. In addition, in women, BMI and fat mass increase during the menopause transition (MT) [21], and it remains to be determined if these MT-related changes in body size and body composition affect substantial change in CRP levels in women. A handful of studies have examined change in body composition from premenopause to postmenopause in relation to contemporaneous change in CRP, and have found conflicting results [22–24].

Although premenopausal CRP levels are inversely associated with serum estradiol levels [25], an analysis of CRP trajectories in the Study of Women’s Health Across the Nation (SWAN) found that CRP levels do *not* go up in every woman during the MT (when serum estradiol levels come down rapidly); instead, some women, especially those who were obese in premenopause, experience declines in CRP levels during the MT [26]. We therefore, hypothesize that increases in CRP levels during the MT are driven primarily by MT-associated increases in BMI and fat mass (Hypothesis 1).

It is now well recognized that BMI does not reflect fat mass well in some race/ethnicity groups. In particular, at the same BMI, Asian women have more and Black and Hispanic women have less fat mass than White women [27,28]. We therefore also hypothesized that increases in fat mass would be associated with increases in CRP levels more strongly and uniformly across race/ethnicity groups than would be changes in BMI (Hypothesis 2).

In postmenopause, fat mass is the primary source of estrogen, via aromatization of adrenally produced testosterone. Since estrogen is thought to protect against inflammation [29,30], we further hypothesized that fat mass increases in postmenopause would be associated with smaller, but non-zero, increases in CRP levels than in premenopause (Hypothesis 3).

To test these hypotheses, we use prospectively collected data from the multi-ethnic SWAN cohort, which includes repeated measurements of body size, body composition, CRP, and important confounders (including financial hardship and medications such as statins and NSAIDs) over more than 2 decades, from before to after the MT. We use individual fixed effects (IFE) analysis, which focuses on the relationship between within-woman changes in predictors and dependent variable, and eliminates all confounding by time-fixed characteristics of the participant (including characteristics that have not been measured). IFE estimates can be interpreted as causal if there are no time-varying confounders [31].

## Methods

SWAN is a multi-center, longitudinal cohort study of the MT. Eligibility criteria at inception were age 42–52 years, having an intact uterus and at least one ovary, having menstruated in the previous three months, not being pregnant and not currently using hormone therapy (HT). Seven study sites enrolled 3,302 women with each site enrolling non-Hispanic White women and women from one minority group: Black (Boston, Chicago, Pittsburgh, southeast Michigan), Chinese (Oakland, California), Japanese (Los Angeles) and Hispanic (Newark, New Jersey). Since the 1996–97 baseline, 15 annual or bi-annual follow-up visits were conducted prior to the pandemic; 74% of surviving women participated in the 15th follow up visit, and every participant had completed the MT.

### Study Samples

To be included in this analysis, women needed to *not* be diagnosed with diabetes or cancer and *not* have taken immune modulator medications prior to having admissible CRP and BMI measurements (i.e., for CRP - not deemed acute inflammation; for BMI – not an outlier observation as defined below) in *at least two* study visits when *not* pregnant and *not* on systemic menopausal hormones or selective estrogen receptor modulator (SERM) therapy. After excluding visits when CRP was not measured (or deemed to reflect acute inflammation, using rules previously developed in the study [32]) and visits for women when they were on systemic hormone therapy (HT) or were missing BMI data, we lost 33 women and were left with 16,591 observations from 3,269 women. We censored 427 observations for new diagnosis of cancer or initiating immunomodulator therapy, and dropped 1 observation when the woman was pregnant, 57 observations when the women were on SERMs, and 52 observations that were missing information on smoking or HT use, leaving us with 16,064 observations from 3,266 women. We dropped women with BMI in the top and bottom 1% of the distribution and censored women when their change in BMI from baseline was in the top or bottom 1%. We excluded 101 women who had diabetes at baseline and censored an additional 926 observations after new diagnosis of diabetes. After this censoring, we lost an additional 705 women who were left with only CRP measurement, resulting in a sample of 2,320 women with 13,100 observations for the primary analysis with BMI (*Study Sample 1*).

Body composition, using dual x-ray absorptiometry (DXA), was measured at 5 of the 7 SWAN study sites. For the analysis with fat mass (in place of BMI), we excluded 579 women and dropped visits without body composition data. Of the remaining women, 60 women who were left with only CRP measurement had to be dropped, leaving us with 1,681 women and 9,385 observations for the fat mass analysis (*Study Sample 2*).

### Measurements

SWAN measured CRP (mg/L) at baseline through follow-up visit 7, and at follow-ups 9, 10, 12, and 15. Over the decades of the SWAN study, CRP assays changed three times to newer assays with lower limits of detection (LLD). Therefore, SWAN cross calibrated the first three assays (an ultrasensitive rate immunonephelo-metric method with LLD of 0.3 mg/L, BN100, Dade-Behring, Marburg, Germany; the ACE UltraWide Range assay, a latex-enhanced turbidimetric in vitro immunoassay with LLD of 0.1 mg/L, Alfa Wassermann, West Caldwell, NJ; a high sensitivity immunoassay with LLD of 0.1 mg/L, Alfa Wassermann, West Caldwell, NJ) to the fourth assay, the Human High Sensitivity CRP ELISA assay (R&D Systems, DCRP00) with LLD of 0.00001 mg/L, using 200 paired samples to create the calibration equations for each of the first 3 assays. After calibration, correlations between the original three CRP assays and the fourth, high-sensitivity ELISA were each > 0.94. In addition, we re-measured CRP in all samples that had values below LLD from the first 3 assays using the fourth assay (high-sensitivity ELISA) on serum samples stored in the SWAN Repository. CRP values that were gauged to be reflective of acute inflammation, using a previously reported algorithm that compared CRP measurements to previous and later measurements in the same woman [32], were set to missing. CRP values were (natural) log-transformed to ln(CRP) for analyses.

Self-defined race/ethnicity [Black, Chinese, Hispanic, Japanese, White] was collected at study baseline; menstrual bleeding patterns, prescription and over-the-counter medication use (including menopausal hormone therapy, corticosteroids, aspirin, and non-steroidal anti-inflammatory drugs: NSAIDs), and smoking status were ascertained using standardized questionnaires and interviews at each study visit. Body height and weight were measured at each visit, and combined to create BMI. We used menstrual histories, acquired at each visit, to create MT stage: Premenopause (regular menses, unchanged from usual), early perimenopause (menses within the prior 3 months but less predictable than prior), late perimenopause (> 3 months but < 12 consecutive months of amenorrhea) and Postmenopause (no menses for > 12 consecutive months). Surgical postmenopause refers to bilateral salpingoophorectomy with or without hysterectomy.

We measured body composition annually using Hologic DXA scanners (Hologic, Inc., Waltham, Massachusetts) at five of the seven SWAN sites. Four of the sites upgraded their scanners during the study and created cross-calibration equations to make measurements comparable across visits, as previously reported [33]. We calculated fat mass index (FMI) as total DXA-measured fat mass (in kg) divided by the square of body height (in meters), analogous to the construction of BMI. This allows a direct comparison of CRP prediction ability between BMI and FMI which are in the same units (kg/m^2^); in addition, FMI is considered a better measure of adiposity than other metrics such as percentage fat mass [34–36].

### Statistical Analysis

We use IFE regression to estimate the association between predictors (MT stage, BMI, FMI) and ln(CRP), and control for time-varying covariates. IFE regression has two significant advantages for clinical inference over other longitudinal methods. First, each participant serves as their own control over repeated measurements. Specifically, regression estimates capture the relationship between within-individual change over time in the predictors and within-individual change in the dependent variable, and do not rely on between-individual comparisons. The second advantage is that IFE eliminates confounding by all individual-specific characteristics that do not change during the study period. This includes confounding by time-fixed variables that are measured (e.g., race/ethnicity, study site), and those that have or cannot be ascertained (e.g., genetics, family history, childhood environment, pre-study health and pre-study lifestyle). These two properties, together, strengthen our ability to interpret estimated associations as the anticipated effects on CRP of changes in a risk factor (MT stage, BMI, FMI in this study) in an individual in the absence of simultaneous changes in factors not included in the model.

To control for confounding by changes over time in other measured variables, we included smoking status and use of oral corticosteroids, statins, aspirin, and NSAIDS as covariates in all IFE models. Ln(CRP) was the dependent variable in all models; MT stage and BMI were the primary predictors in the first model (in Study Sample 1). To allow for possible non-linearity in the BMI-CRP relationship, we first examined LOESS plots of ln(CRP) at the baseline visit as a function of baseline BMI, after dropping the bottom and top 1% of the data distribution. This demonstrated a piece-wise linear relationship with one reduction in slope at around BMI of 34 kg/m^2^ (Fig. 1), suggesting that change in BMI has a smaller association with change in CRP when BMI is above this threshold. We, therefore, included an interaction term between current BMI (time-varying) and a time-fixed indicator variable for women whose average BMI (over the study) exceeded 34 kg/m^2^.

Because FMI was measured only in a subset of women (who were in Study Sample 2), we re-ran the model with primary predictors BMI and MT in Study Sample 2, to confirm that selection bias did not impact findings in the smaller sample. We then added interactions of BMI (time-varying) with race/ethnicity (time-fixed) to test for differential associations of BMI with CRP in the different racial/ethnic groups.

In the second analysis in Study Sample 2, MT stage and FMI were the primary predictors. As with BMI, we first examined LOESS plots of ln(CRP) at the baseline visit as a function of baseline FMI. This also suggested a piece-wise linear relationship, but with reduction in slope at around FMI of 15 kg/m^2^ (Fig. 2). We, therefore, included an interaction term between current FMI (time-varying) and an indicator variable (time-fixed) for women whose average FMI (over the study) exceeded 15 kg/m^2^. In a separate step, parallel to the BMI analyses, we added interactions of FMI (time-varying) with race/ethnicity (time-fixed) to test for differential associations of FMI with CRP in the different racial/ethnic groups.

Finally, to test for differential associations of fat mass change over the various stages of the MT, we included an interaction of FMI with MT stage.

## Results

Study samples 1 and 2 were similar to the entire SWAN sample with respect to major characteristics of interest, with a few expected exceptions (Table 1). Since Hispanic women were seen only at one of the two SWAN sites that did not collect body composition data, there were no Hispanic women in Study Sample 2. BMI, FMI, and CRP were smaller in our study samples than in the full SWAN cohort, because the study samples excluded women with diabetes who tend to be heavier, have larger fat mass, and more chronic inflammation. The lower uptake of statin therapy in the study samples also reflects the same exclusion, since individuals with diabetes are likely to be taking statins.

Average age at the baseline visit was 46 years, around half the women in the study sample were White, one in four were Black, one in seven were Japanese, and one in eight were Chinese. More than half the women in the study sample were premenopausal at baseline, average BMI at baseline was around 27 kg/m^2^, and average baseline FMI was 10 kg/m^2^. Median CRP level in the serum at the baseline visit was 1.4 mg/L. Median number of study visits was 8, but a quarter of the sample had 10 or more visits. Just under one in three visits were in postmenopause.

### Adjusted Associations with BMI Change

In IFE analysis, adjusted for time-varying covariates (smoking, use of oral steroids, NSAIDs, aspirin, and statins, and MT stage), in women whose average BMI over the study was 34 kg/m^2^ or less, each kg/m^2^ BMI increase over time was associated with 0.147 increment in natural log-transformed CRP, or 15.8% increment in CRP, 95% confidence interval, (14.4%, 17.1%). The interaction with the indicator for BMI exceeding 34 was statistically significant, so that in women with study-average BMI greater than 34 kg/m^2^, each kg/m^2^ increase in BMI was associated with only 0.086 increase in ln CRP, or 9.0% increase in serum CRP. Consistent with the anti-inflammatory effects of statins, initiation of statin therapy was associated with a significant reduction in CRP level. However, change in MT stage had no association with serum CRP level, independent of change in BMI.

These associations persisted with only small changes in magnitude when the analyses were repeated in the smaller Study Sample 2 – See Table 2. Race/ethnicity also had a significant interaction with BMI, so that change over time in BMI had a smaller association with change in CRP in Black women than in White women. In White women with study-average BMI no greater than 34 kg/m^2^, each kg/m^2^ BMI increase over time was associated with 0.166 increase in ln(CRP) or 18.1% increase in CRP, while in similar Black women ln(CRP) increased by only 0.112 and CRP by only 11.9%. In both Chinese and Japanese women, the BMI association with CRP was statistically similar to that in White women (Table 2).

### Adjusted Associations with FMI Change

Adjusted for the same time-varying covariates in IFE analyses, within-woman change over time in FMI had larger associations with change over time in serum CRP (Table 3). In women whose average FMI over the study was 15 kg/m^2^ or less, each kg/m^2^ increment in FMI was associated with 0.204 increase in ln(CRP), which translates to 22.6% increase in CRP, 95% confidence interval (20.3%, 24.9%). The interaction with the indicator for FMI exceeding 15 was statistically significant, so that in women with study-average FMI greater than 15 kg/m^2^, each kg/m^2^ increase in FMI was associated with only 0.119 increase in ln CRP, or 12.6% increase in serum CRP. Unlike with BMI, race/ethnicity did not have statistically significant interactions with FMI, so that the association of FMI change with CRP change was similar in all four race/ethnicity groups (Table 3).

With the addition of an interaction of FMI with MT stage, although change in MT stage was itself *not* associated with CRP change, MT stage did statistically modify the association of FMI change with CRP change, so that the association was statistically significantly smaller in both natural and surgical postmenopause. In women with study-average FMI no greater than 15 kg/m^2^, while each kg/m^2^ FMI increase over time in premenopause is associated with 0.223 increase in ln(CRP) or 25.0% increase in CRP, the corresponding increases in ln(CRP) and CRP are only 0.198 and 21.9% in natural postmenopause and 0.151 and 16.3% in surgical postmenopause.

## Discussion

As hypothesized, this longitudinal analysis of repeated measurements of serum CRP levels in a cohort of midlife women who transitioned from pre to postmenopause found that 1) Increase in fat mass is the primary driver of CRP increase, while the MT does not have an independent *direct* association with CRP; 2) BMI associations with CRP vary by race/ethnicity, while fat mass associations with CRP do not vary significantly by race/ethnicity, consistent with previous findings that BMI reflects fat mass differently in different race/ethnicity groups; 3) The association of fat mass with CRP is statistically smaller in postmenopause (when fat mass is the primary source of circulating estrogen) than it is in premenopause, consistent with an anti-inflammatory effect of estrogen generated in adipose tissue. However, the size of this ‘benefit’ is modest at best: 21.8% increase in CRP in postmenopause per kg/m^2^ increase in FMI compared to 24.1% CRP increase in early perimenopause.

At least one previous study in 69 women observed once before and once after the MT, separated by a mean of 4.1 years, found no MT effect but did find a significant positive correlation between change in intraabdominal fat and change in CRP [22]. One other small study in 103 White women observed once before and after the MT, 3–7 years apart, did find an MT effect: CRP went up by an average of 0.4 mg/dL; however, this analysis did not control for increases in body weight or fat mass [24]. A larger study in 1,492 women observed once before the MT and once, 2.5 years later, after the MT, also found no association between the MT and CRP [23]. Our study is the first study that uses multiple repeated measurements, before, during, and after the MT, in a large, multi-ethnic cohort to provide strong evidence of a direct effect of the menopause transition on circulating CRP levels, independent of changes in body weight or body composition, controlling for all time-invariant factors that may have confounded the estimated relationships.

We show that any changes in CRP level over the MT are primarily related to changes in fat mass. We also show that the association of fat mass with CRP is not different between the four race/ethnicity groups studied here. While the association of BMI with CRP did vary by race/ethnicity, this effect modification can be attributed to previously documented race/ethnicity differences in the relationship between fat mass and BMI. Thus, Black women who on average have less fat mass than White women with the same BMI, have a smaller BMI-CRP association but the FMI-CRP association is statistically no different in Black vs. White women. The third new finding from this study is that the FMI-CRP association is modestly smaller in postmenopause than in premenopause, probably because fat mass is the primary source of estrogen in postmenopause and estrogen has known anti-inflammatory effects [29,30]. However, the inflammatory effects of fat mass appear to be much larger than any protection conferred by estrogen.

The major limitation of this study is its observational design, which limits causal inference. We maximize our ability to make causal inferences by using individual fixed effects analyses, which eliminates confounding by all time-fixed characteristics, including those not observable and pre-study covariates such as health and lifestyle choices. However, this approach does not remove confounding by individual characteristics that change during the study and are not explicitly included in the regression models. Secondly, generalizability of our findings is limited by the underrepresentation in the SWAN cohort of Hispanic women and their non-participation in body composition measurements and the exclusion of women with premature menopause and pre/peri-menopausal hysterectomy from the cohort. Study strengths include 17 waves of longitudinal data collection over 20 + years across the menopause transition in a large multi-ethnic cohort, with more than 10,000 repeated measurements of CRP, and the use of individual fixed effects analysis.

## Conclusions

In summary, this large longitudinal study conducted over a 20-year time span showed definitively that midlife changes in CRP in women are driven primarily by changes in fat mass and not directly by the MT. Each kg/m^2^ decrease in fat mass index appears to reduce CRP levels nearly as well as initiating statin therapy regardless of race/ethnicity, and the effect smaller in postmenopause than before the MT. We conclude that intensive lifestyle changes in midlife and early initiation of statin therapy may be effective, potentially additive, interventions to prevent long term consequences of chronic inflammation, that should be tested in clinical trials.

## Figures and Tables

**Figure 1:**
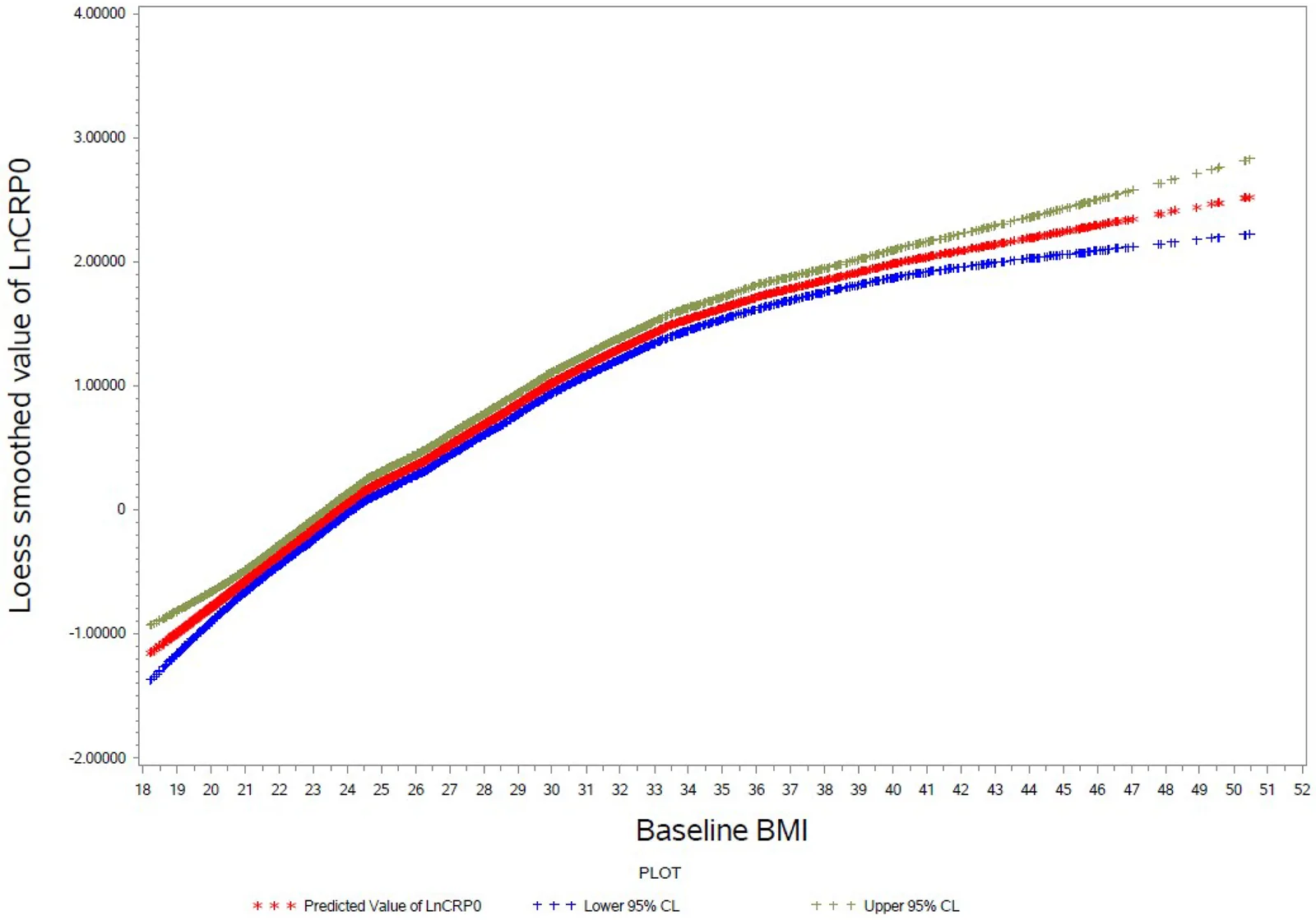
LOESS-smoothed plot of baseline C-reactive protein level as function of body mass index at baseline

**Figure 2:**
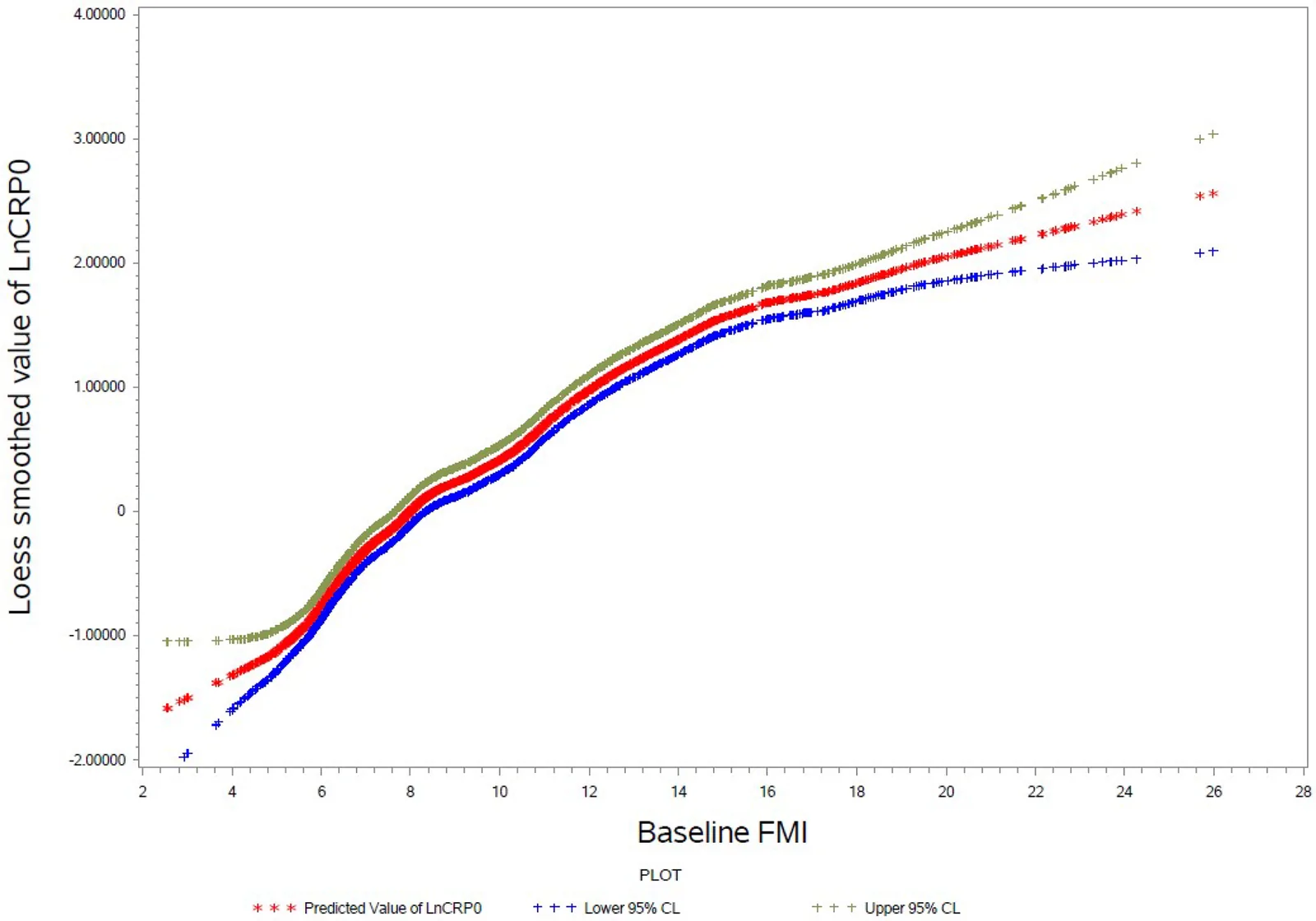
LOESS-smoothed plot of baseline C-reactive protein level as function of fat mass index at baseline

**Table 1:** Descriptive Statistics^1^ of Study Samples’ Characteristics at Study Baseline and over Repeated Observations

At Study Baseline	SWAN Cohort (N = 3,302)	Study Sample 1 (N = 2,320)	Study Sample 2 (N = 1,681)
Age (years)	46 (3)	46 (3)	46 (3)
Race/Ethnicity Black	934 (28%)	611 (26%)	419 (25%)
White	1551 (47%)	1096 (47%)	827 (49%)
Chinese	250 (8%)	213 (9%)	207 (12%)
Japanese	281 (9%)	237 (10%)	228 (14%)
Hispanic	286 (9%)	163 (7%)	-
Menopause status Premenopause	1770 (54%)	1313 (57%)	935 (56%)
Early perimenopause	1501 (46%)	986 (43%)	721 (43%)
Current smoker	569 (17%)	341 (15%)	228 (14%)
Body mass index (kg/m^2^)	28.4 (7.3)	27.5 (6.3)	27.0 (6.1)
Fat mass index (kg/m^2^)	10.1 (4.1)	9.8 (3.8)	10.0 (4.0)
C-reactive protein (mg/L)	1.9 (0.6, 5.9)	1.5 (0.5, 4.5)	1.4 [0.5, 4.1]
Ln (C-reactive protein, mg/L)	0.7 (1.5)	0.5 (1.4)	0.3 (1.4)
Over Repeated Visits	SWAN Cohort (n = 40,150)	Study Sample 1 (n = 13,100)	Study Sample 2 (n = 9,385)
Number of study visits	16 [14, 16]	8 [5, 10]	8 [5, 10]
Postmenopause	20700 (52%)	3928 (30%)	2844 (30%)
Current smoker	4354 (13%)	1548 (12%)	1007 (11%)
On oral steroids	1193 (3%)	318 (2%)	239 (3%)
On NSAIDs	10820 (27%)	3779 (29%)	2853 (30%)
On aspirin	5191 (13%)	1396 (11%)	990 (11%)
On statins	4331 (11%)	762 (6%)	528 (6%)
Body mass index (kg/m^2^)	28.9 (7.3)	27.8 (6.3)	27.3 (6.0)
- change from baseline	0.85 (2.59)	0.70 (1.74)	0.66 (1.69)
Fat mass index (kg/m^2^)	10.9 (4.4)	10.4 (4.0)	10.3 (4.0)
- change from baseline	0.75 (1.71)	0.62 (1.24)	0.62 (1.24)
C-reactive protein (mg/L)	1.9 (0.6, 5.5)	1.5 [0.5, 4.4]	1.4 [0.5, 4.0]
Ln (C-reactive protein, mg/L)	0.6 (1.5)	0.4 (1.4)	0.3 (1.4)
- change from baseline	0.02 (0.98)	0.05 (0.97)	0.04 (0.99)

Fat mass index = total fat mass divided by square of height; ln = Natural log; N = Sample size; n = Number of repeated observations; SWAN = Study of Women’s Health Across the Nation;

Study Sample 1: Analytic sample for estimating the association of within-woman change in body mass index with within-woman change in C-reactive protein. See text for inclusion criteria.

Study Sample 2: Analytic sample for estimating the association of within-woman change in total fat mass with within-woman change in C-reactive protein. See text for inclusion criteria.

1 Mean (standard deviation) or median [interquartile interval] or number (%).

**Table 2:** Adjusted Association^1^ of within-woman change in body mass index with change in log-transformed C-reactive protein

	*Study Sample 1*	*Study Sample 2*	
	Base model	Base model	With race/ethnicity interactions
Current smoker	7.1 (−2.9, 17.1)	9.6 (−2.9, 22.2)	9.3 (−3.2, 21.9)
On oral steroids	−0.4 (−10.3, 9.5)	2.8 (−8.6, 14.3)	3.2 (−8.3, 14.6)
On NSAIDs	2.9 (−0.7, 6.4)	2.8 (−1.4, 7.1)	2.8 (−1.4, 7.1)
On aspirin	−1.9 (−7.1, 3.2)	−4.5 (−10.8, 1.8)	−4.5 (−10.7, 1.8)
On statins	**−25.7** (−32.7, −18.6)	**−28.7** (−37.5, −19.9)	**−29.1** (−37.9, −20.3)
Menopause (ref: Premenopause)			
Early perimenopause	0.9 (−3.4, 5.1)	0.1 (−5.0, 5.2)	0.0 (−5.1, 5.1)
Late perimenopause	−3.6 (−9.7, 2.5)	−2.3 (−9.5, 4.9)	−2.3 (−9.5, 4.9)
Natural postmenopause	−2.0 (−6.8, 2.8)	−3.1 (−8.8, 2.6)	−3.2 (−8.9, 2.5)
Surgical postmenopause	15.9 (−8.4, 40.3)	21.0 (−10.7, 52.8)	22.4 (−9.4, 54.2)
Body mass index (kg/m^2^)	**14.7** (13.5, 15.8)	**15.7** (14.3, 17.1)	**16.6** (14.8, 18.4)
Effect modification if	**−6.1** (−8.4, −3.8)	**−6.0** (−8.9, −3.1)	**−5.4** (−8.4, −2.4)
BMI > 34 kg/m^2^			
Black	-	-	**−3.1** (−5.9, −0.3)
Chinese	-	-	−2.8 (−7.8, 2.2)
Japanese			−0.5 (−3.8, 4.9)

BMI = body mass index; FMI = Fat mass index = total fat mass divided by square of height

Study Sample 1: Analytic sample for estimating the association of within-woman change in body mass index with within-woman change in C-reactive protein. See text for inclusion criteria.

Study Sample 2: Analytic sample for estimating the association of within-woman change in total fat mass with within-woman change in C-reactive protein. See text for inclusion criteria.

1 Associations are estimates from individual fixed effects models. They are listed as point estimate (95% confidence interval) scaled by 100. If smaller than 5, the scaled values can be interpreted as % change in CRP per unit increment of the predictor. Bolded point estimates are statistically significant (p < 0.05) and italicized point estimates are marginally statistically significant (p < 0.1 but > 0.05). Time-fixed characteristics (such as race/ethnicity, study site) are implicitly controlled in individual fixed effects models.

**Table 3:** Adjusted Association^1^ of within-woman change in fat mass index with change in log-transformed C-reactive protein

Current smoker	Base model	With race/ethnicity interactions	+ menopause status interactions
	9.1 (−3.4, 21.7)	8.7 (−3.9, 21.3)	8.7 (−3.9, 21.3)
On oral steroids	3.1 (−8.3, 5.8)	3.3 (−8.1, 14.8)	3.3 (−8.1, 14.8)
On NSAIDs	3.4 (−0.9, 7.7)	3.4 (−0.8, 7.7)	3.2 (−1.0, 7.5)
On aspirin	−5.0 (−11.2, 1.3)	−5.0 (−11.2, 1.3)	−4.8 (−11.1, 1.5)
On statins	**−25.2** (−34.0, −16.4)	**−25.3** (−34.1, −16.6)	**−24.4** (−33.2, −15.6)
Menopause (ref: Premenopause)			
Early perimenopause	−0.4 (−5.5, 4.7)	−0.4 (−5.5, 4.7)	−1.1 (−6.2, 4.0)
Late perimenopause	−4.8 (−12.0, 2.5)	−4.8 (−12.0, 2.6)	−7.1 (−14.5, 0.2)
Natural postmenopause	−5.1 (−10.9, 0.6)	−5.2 (−11.0, 0.5)	−5.3 (−11.1, 0.4)
Surgical postmenopause	9.5 (−22.4, 41.3)	11.2 (−20.7, 43.1)	23.0 (−11.8, 57.9)
Fat mass index (kg/m^2^)	**20.4** (18.5, 22.2)	**21.2** (18.9, 23.5)	**22.3** (19.8, 24.8)
Effect modification if	**−8.5** (−12.5, −4.3)	**−7.9** (−12.1, −3.7)	**−6.7** (−10.9, −2.5)
FMI >15 kg/m^2^			
Black	-	−3.0 (−6.7, 0.7)	−3.1 (−6.8, 0.6)
Chinese	-	−0.8 (−7.5, 5.8)	−1.3 (−7.9, 5.3)
Japanese	-	−0.4 (−6.3, 5.6)	−0.0 (−6.0, 5.9)
Early perimenopause	-	-	−0.6 (−2.0, 0.7)
Late perimenopause	-	-	0.5 (−1.3, 2.3)
Natural postmenopause	-	-	**−2.5** (−4.0, −1.0)
Surgical postmenopause	-	-	**−7.2** (−14.5, −0.02)

BMI = body mass index; FMI = Fat mass index = total fat mass divided by square of height

1 Associations are estimates from individual fixed effects models. They are listed as point estimate (95% confidence interval) scaled by 100. If smaller than 5, the scaled values can be interpreted as % change in CRP per unit increment of the predictor. Bolded point estimates are statistically significant (p < 0.05) and italicized point estimates are marginally statistically significant (p < 0.1 but > 0.05). Time-fixed characteristics (such as race/ethnicity, study site) are implicitly controlled in individual fixed effects models. Menopause status effects in the full model (last column) are listed for a woman whose FMI is 10 kg/m^2^.

## Data Availability

Datasets from SWAN are publicly available at the study website (http://www.swanstudy.org/swanresearch/data-access/) and at the National Institute of Aging repository (https://agingresearchbiobank.nia.nih.gov/). To preserve participant confidentiality, not all of the data collected are included in the public use datasets. Investigators who require assistance accessing the public use dataset may contact the SWAN Coordinating Center at the following email address: swanaccess@edc.pitt.edu.

## Abbreviations

BMI: Body Mass Index
CRP: C-reactive Protein
FMI: Fat Mass Index
HT: Hormone Therapy
IFE: Individual Fixed Effects
LLD: Lower Limit of Detection
MT: Menopause Transition
SERM: Selective Estrogen Receptor Modulator
SWAN: Study of Women’s Health Across the Nation
